# Shift work, headache, and migraine in a large cohort of Dutch nurses

**DOI:** 10.1093/annweh/wxag080

**Published:** 2026-09-16

**Authors:** Tara van der Grinten, Sylvia H J Jochems, Flora van Leeuwen, Michael Schaapveld, Hans Kromhout, Roel Vermeulen, Jelle Vlaanderen, Susan Peters

**Affiliations:** Institute for Risk Assessment Sciences, Utrecht University, PO Box 80178, 3508 TD Utrecht, The Netherlands; Institute for Risk Assessment Sciences, Utrecht University, PO Box 80178, 3508 TD Utrecht, The Netherlands; Department of Epidemiology, Netherlands Cancer Institute, Plesmanlaan 121, 1066 CX Amsterdam, The Netherlands; Department of Epidemiology, Netherlands Cancer Institute, Plesmanlaan 121, 1066 CX Amsterdam, The Netherlands; Institute for Risk Assessment Sciences, Utrecht University, PO Box 80178, 3508 TD Utrecht, The Netherlands; Institute for Risk Assessment Sciences, Utrecht University, PO Box 80178, 3508 TD Utrecht, The Netherlands; Institute for Risk Assessment Sciences, Utrecht University, PO Box 80178, 3508 TD Utrecht, The Netherlands; Institute for Risk Assessment Sciences, Utrecht University, PO Box 80178, 3508 TD Utrecht, The Netherlands

**Keywords:** headache, migraine, shift work, occupational health

## Abstract

**Objective:**

Shift work is common in healthcare and may contribute to headache disorders through mechanisms such as circadian disruption and sleep disturbance. However, evidence from large prospective cohort studies remains limited. This study investigated the associations between shift work and headache and migraine in a cohort of Dutch nurses.

**Methods:**

Participants completed questionnaires in 2011 (*n* = 59,768) and 2017 (*n* = 37,713), reporting headache, migraine, work history, sleep problems, and lifestyle factors. Headache and migraine were assessed using the validated Headache Impact Test (HIT-6) and ID-Migraine questionnaires. Associations between shift work and the prevalence of headache or migraine at baseline and follow-up were examined using Poisson regression with robust variance to directly estimate relative risks. Additional analyses examined current shift work status and shift work duration.

**Results:**

Ever shift work was associated with a higher prevalence of headache at baseline (relative risk = 1.04, 95% confidence interval: 1.01 to 1.07), although this association attenuated after adjustment for sleep problems (relative risk = 1.01, 95% confidence interval: 0.98 to 1.04). For migraine, ever shift work was associated with a higher prevalence at follow-up (relative risk = 1.13, 95% confidence interval: 1.02 to 1.26), whereas no association was found at baseline. Current shift work was not consistently associated with headache or migraine.

**Conclusions:**

Ever shift work was associated with a small increase in the prevalence of migraine. Associations with headache attenuated after adjustment for sleep problems. No consistent associations were observed for current shift work.

What's important about this paper Shift work is a common feature of healthcare work, yet its relationship with headache disorders remains incompletely understood. Among a cohort of nurses, this study found ever having shift work was more strongly associated with migraine than with headache, and the association with headache was attenuated after adjustment for sleep. These findings contribute to the evidence base needed to guide future occupational health research and preventive strategies for shift workers.

## Introduction

According to the European Working Conditions Survey, around 21% of workers in the EU work shifts (Parent-Thirion et al. 2017). Shift work refers to working outside the standard 9 AM to 5 PM schedule, with night shift work involving at least 2 working hours between 10 PM and 5 AM (Pijpe et al. 2014). Evidence suggests a possible association between shift work and headache (Wang et al. 2024).

Shift workers are exposed to a complex range of factors, including irregular work schedules, changes in diet, exposure to artificial light at night, and social disruption (van der Grinten et al. 2025). These exposures may contribute to stress, physical inactivity, and sleep disturbances, which have been associated with headache symptoms (Scher et al. 2002; Leso et al. 2020). Headaches can lead to reduced quality of life and productivity (Cavallini et al. 1995; Selekler et al. 2015). This is particularly concerning in essential fields like nursing, where staffing shortages already pose serious challenges.

Around 18% of women and 8% of men in the Netherlands report suffering from migraines or severe headaches (Statistics Netherlands 2026). Previous research has shown that migraines are 3 to 4 times more common in adult women than men (Tonini 2018). Women also have a higher risk of headaches in general (Stovner and Andree 2010). Since nursing often involves shift work and predominantly employs women, the impact of shift work-related headaches may be particularly relevant in this field.

Research on the association between shift work and headache disorders remains inconsistent. Some cross-sectional studies have reported higher odds of headache and migraine among shift workers, whereas others report no clear association (Jakobsen et al. 2017; Bjorvatn et al. 2018). Longitudinal evidence is limited but suggests a possible association between shift work-related exposures and headache outcomes (Appel et al. 2020; Kristoffersen et al. 2024). Reviews of the literature likewise indicate a possible association, while emphasizing substantial heterogeneity in exposure definitions, outcome assessment, and study quality (Leso et al. 2020; Wang et al. 2024). Overall, stronger data are needed to clarify the association between shift work and headache and migraine.

In addition to investigating the association between shift work and headaches, it is important to identify which characteristics of shift work are associated with headache outcomes, as this may provide insight into potential mechanisms. Since shift workers regularly work outside of conventional daytime hours and often have rotating shifts, they may experience long-term circadian disruption, disrupted sleeping and eating patterns, and related lifestyle changes, which may contribute to headache and migraine (Borkum 2016; Richter et al. 2021). We hypothesized that shift work would be associated with headache and migraine and explored whether factors such as sleep disturbances may play a role in this association.

In this study, we investigated differences in headache and migraine prevalence between shift and day workers in a cohort of Dutch nurses. We compared the prevalence of self-reported headaches and migraine between shift and day workers at baseline and at 6-yr follow-up and evaluated whether changes in shift work status between the two waves were associated with headache development and recovery. We hypothesized that shift workers would have a higher prevalence of headache and migraine than permanent day workers. Additionally, we examined whether adjustment for sleep problems influenced the observed associations.

## Methods

### Study population

Data were collected as part of the Nightingale cohort study, as previously described by Pijpe et al. (2014). Briefly, 59,947 participants completed a web-based questionnaire in 2011 covering work history, shift work characteristics, health status, medication use, sleep patterns, and lifestyle factors such as physical activity and alcohol consumption. The questionnaire also included specific questions about headache (Headache Impact Test, HIT-6) and migraine (ID-Migraine) (Lipton et al. 2003; Yang et al. 2011). In addition, participants were asked whether they had received a physician diagnosis of migraine.

Participants received a follow-up questionnaire in 2017, which was completed by 37,730 respondents. The follow-up questionnaire contained similar questions to the baseline questionnaire, including the HIT-6 and ID-Migraine sections. After excluding participants with missing information on lifetime shift work status, the analytical populations consisted of 59,768 participants at baseline and 37,713 participants at follow-up.

### Shift work

Shift work exposure was assessed in both baseline and follow-up questionnaires. The questionnaires collected detailed information on participants’ occupational history and shift work characteristics, including early, evening, night, and sleep shifts, as well as shift timing, frequency, consecutive shifts, and rotation schedules. Shift work was recorded for periods of at least 6 months.

For this study, we used 4 exposure variables: lifetime shift work status (ever versus never shift work), duration of shift work, current shift work status, and changes in current shift work status between baseline and follow-up. Current shift work status and changes in current shift work status were based on the questionnaire items on current night shift work.

The occupational history additionally recorded job title, sector, start and stop years, working hours, and detailed shift work characteristics for each job, including shift start and end times, number of shifts worked per month, number of consecutive shifts, and rotation schedule (permanent, forward rotating, backward rotating, or constantly changing).

### Headache, migraine, and sleep disturbances

Headache impact was assessed using the HIT-6, which evaluates headache burden during the past 4 weeks (Yang et al. 2011; Table S1). Response options to each of the 6 questions were never, rarely, sometimes, very often, or always. These responses were scored as 6, 8, 10, 11, and 13 points, respectively. Total scores ≤49 were categorized as no headache, scores of 50 to 55 as light headache, 56 to 59 as moderate headache, and ≥60 as severe headache.

Migraine was assessed using the ID-Migraine questionnaire (Lipton et al. 2003), which consists of 3 yes/no questions on migraine symptoms during the past 3 months (Table S2). Participants received one point for each affirmative response, and those scoring ≥2 points were classified as having migraine. Among participants classified as having headache, those who did not complete the ID-Migraine questions were considered missing for the migraine analyses.

Sleep disturbances were assessed using the Sleep Problems Index II from the Medical Outcomes Study (MOS) Sleep Scale (Hays and Stewart 1992). The MOS Sleep Scale includes items assessing sleep initiation, sleep maintenance, sleep quantity, sleep adequacy, somnolence, and respiratory problems during sleep. Responses are recorded on a 6-point Likert scale across 12 items, after which scores are converted to a scale ranging from 0 to 100, with higher scores indicating more sleep problems. Sleep problems were assessed during the past 4 weeks.

### Statistical analyses

Descriptive statistics are presented as percentages for categorical variables and medians with interquartile ranges (IQRs) for continuous variables. Associations between shift work and the prevalence of headache or migraine at baseline, follow-up, and in the combined analyses were estimated using Poisson regression with robust variance to directly estimate relative risks (RRs) and corresponding 95% confidence intervals (CIs). These analyses evaluated the prevalence of headache and migraine at each assessment. Headache was modeled as headache (HIT-6 ≥ 50; light, moderate, or severe) versus no headache (HIT-6 ≤ 49). Migraine was modeled as migraine (ID-Migraine ≥2 points) versus no migraine (≤1 point). These binary outcomes were defined a priori based on established cut-points of validated instruments and allowed clinically interpretable case definitions (van Duijne et al. 2024; de Bruijn et al. 2025).

For analyses combining baseline and follow-up observations, Poisson regression models with robust variance were fitted while accounting for repeated observations within individuals using clustered standard errors.

The primary analyses evaluated ever-versus-never shift work as the exposure variable. Age was selected as the primary confounder based on a directed acyclic graph (Fig. S1) and was modeled using natural cubic splines.

Models were specified a priori. Model 1 was adjusted for age. Model 2 was additionally adjusted for the Sleep Problems Index II because sleep disturbances have been proposed as a potential mechanism linking shift work and headache disorders. Additional exploratory models further adjusting for alcohol consumption, physical activity, painkiller use, and menopausal status were fitted to evaluate the robustness of the findings. As these adjustments did not materially change the estimates, these models are not presented.

Additional analyses examined shift work duration (never, <10 yr, 10 to 19 yr, and ≥20 yr) and current shift work status as exposure variables. Shift work duration models were adjusted for age. Models evaluating current shift work status followed the same adjustment strategy as the primary analyses, with Model 1 adjusted for age and Model 2 additionally adjusted for the Sleep Problems Index II.

Because only 2 questionnaire waves were available (2011 and 2017), longitudinal analyses were restricted to comparisons between baseline and follow-up.

To investigate changes in headache status between baseline and follow-up, headache recovery and headache development were analyzed according to changes in current shift work status. Recovery was defined as a transition from headache (HIT-6 ≥ 50) at baseline to no headache at follow-up, whereas development was defined as a transition from no headache at baseline to headache at follow-up. Poisson regression with robust variance was used to estimate RRs for transitions in headache status (recovery and development) according to changes in current shift work status.

To investigate whether the association between shift work and headache at follow-up differed according to baseline headache status, participants were stratified into those with and without headache at baseline, and separate models were fitted within each stratum. Similar analyses were performed for migraine.

All analyses were conducted using complete-case analysis. All statistical analyses were performed using R version 4.4.0.

## Results

A total of 59,768 nurses were included in the baseline analyses, of whom 37,713 also completed the follow-up questionnaire. The median age at baseline was 48.0 yr (IQR: 39 to 55). Among participants who completed both questionnaires, the median age at baseline was 49.0 yr (IQR: 42 to 56), increasing to 55.7 yr (IQR: 49 to 63) at follow-up.

Among baseline participants, 81.4% had ever worked shifts, compared with 84.4% among participants who completed the follow-up questionnaire. Current shift work was reported by 20.7% of participants at baseline and 23.1% at follow-up. Among participants who had ever worked shifts, the median duration of shift work was 10 yr (IQR: 5 to 17) at baseline and 12 yr (IQR: 7 to 20) at follow-up.

At baseline, 29.7% of participants reported headache, including 10.4% with severe headache. At follow-up, 24.2% reported headache, including 7.7% with severe headache. Migraine prevalence was 11.9% at baseline and 8.8% at follow-up. Participant characteristics are presented in Table 1.

**Table 1:** Baseline and follow-up characteristics of the study population.

	Baseline participants	Baseline participants with follow-up^a^	Follow-up participants
**Age, years, median (IQR)**	48.0 (16.0)	49.0 (14.0)	55.7 (14.4)
**Ever shift work, *n* (%)**	48,649 (81.4%)	31,375 (83.2%)	31,846 (84.4%)
**Never shift work, *n* (%)**	11,119 (18.6%)	6,338 (16.8%)	4,485 (11.9%)
**Lifetime shift work duration, median (IQR)**	10.0 (12.0)	10.0 (12.0)	12.0 (13.0)
**Frequency of night shifts per month, median (IQR)**	5.0 (4.0)	5.0 (3.0)	4.0 (3.0)
**Never shift work, *n* (%)**	11,119 (18.6%)	6,338 (16.8%)	4,485 (11.9%)
** <10 yr**	15,929 (26.7%)	10,055 (26.7%)	6,976 (18.5%)
** 10 to 19 yr**	10,251 (17.2%)	6,870 (18.2%)	6,268 (16.6%)
** ≥20 yr**	6,137 (10.3%)	4,432 (11.8%)	4,788 (12.7%)
** Missing**	16,361 (27.4%)	10,018 (26.6%)	15,196 (40.3%)
**Current shift work, *n* (%)**			
** No**	31,598 (52.8%)	19,989 (53.0%)	21,638 (57.4%)
** Yes**	12,360 (20.7%)	8,011 (21.2%)	8,702 (23.1%)
** Missing**	15,839 (26.5%)	9,713 (25.8%)	7,373 (19.5%)
**Headache, *n* (%)**			
** No headache**	37,613 (62.9%)	24,797 (65.6%)	23,948 (63.5%)
** Light**	7,023 (11.8%)	4,413 (11.7%)	3,860 (10.2%)
** Moderate**	4,502 (7.5%)	2,712 (7.2%)	2,366 (6.3%)
** Severe**	6,210 (10.4%)	3,743 (9.9%)	2,922 (7.7%)
** Missing**	4,449 (7.4%)	2,048 (5.4%)	4,617 (12.2%)
**Migraine, *n* (%)**			
** No**	48,384 (81.0%)	31,345 (83.1%)	30,212 (80.1%)
** Yes**	7,109 (11.9%)	4,409 (11.7%)	3,304 (8.8%)
** Missing**	4,304 (7.2%)	1,959 (5.2%)	4,197 (11.1%)
**Migraine diagnosis, *n* (%)**			
** No**	53,764 (89.9%)	33,810 (89.7%)	30,001 (79.6%)
** Yes**	6,033 (10.1%)	3,903 (10.3%)	3,060 (8.1%)
** Missing**	0	0	4,652 (12.3%)

IQR, interquartile range.

^a^Presents baseline characteristics of participants who also completed the follow-up questionnaire.

### Ever shift work

Age-adjusted analyses showed a slightly higher prevalence of headache among participants who had ever worked shifts compared with permanent day workers (combined RR = 1.04, 95% CI: 1.01 to 1.08). Similar estimates were observed at baseline (RR = 1.04, 95% CI: 1.01 to 1.07) and follow-up (RR = 1.05, 95% CI: 0.99 to 1.10). After additional adjustment for sleep problems, the associations attenuated (Table 2).

**Table 2:** Relative risks for headache according to ever shift work and cumulative shift work duration estimated using Poisson regression with robust variance.

	Baseline questionnaire (2011)	Follow-up questionnaire (2017)	Combined
	RR (95% CI)	RR (95% CI)	RR (95% CI)
*Ever shift work*			
Model 1^a^			
Never shift work	1.00	1.00	1.00
Ever shift work	1.04 (1.01 to 1.07)	1.05 (0.99 to 1.10)	1.04 (1.01 to 1.08)
Model 2^b^			
Never shift work	1.00	1.00	1.00
Ever shift work	1.01 (0.98 to 1.04)	1.02 (0.97 to 1.08)	1.02 (0.99 to 1.05)
*Cumulative shift work duration*			
Model 3^a^			
Never shift work	1.00	1.00	1.00
<10 yr	1.05 (1.01 to 1.08)	1.04 (0.98 to 1.10)	1.04 (1.01 to 1.08)
10 to 19 yr	1.04 (1.00 to 1.09)	1.02 (0.96 to 1.08)	1.03 (0.99 to 1.07)
≥20 yr	1.01 (0.96 to 1.06)	0.98 (0.92 to 1.05)	1.01 (0.96 to 1.06)

RR, relative risk.

Headache was dichotomized into headache (light, moderate, or severe) versus no headache.

^a^Adjusted for age.

^b^Adjusted for age and Sleep Problems Index II.

For migraine, ever shift work was associated with a higher prevalence at follow-up (RR = 1.13, 95% CI: 1.02 to 1.26), whereas little association was observed at baseline. Additional adjustment for sleep problems attenuated the combined estimates, whereas the association at follow-up changed only marginally (Table 3).

**Table 3:** Relative risks for migraine according to ever shift work and cumulative shift work duration estimated using Poisson regression with robust variance.

	Baseline questionnaire (2011)	Follow-up questionnaire (2017)	Combined
	RR (95% CI)	RR (95% CI)	RR (95% CI)
*Ever shift work*			
Model 1^a^			
Never shift work	1.00	1.00	1.00
Ever shift work	1.05 (0.99 to 1.11)	1.16 (1.05 to 1.29)	1.08 (1.02 to 1.14)
Model 2^b^			
Never shift work	1.00	1.00	1.00
Ever shift work	1.00 (0.94 to 1.06)	1.13 (1.02 to 1.26)	1.03 (0.98 to 1.09)
*Cumulative shift work duration*			
Model 3^a^			
Never shift work	1.00	1.00	1.00
<10 yr	1.06 (0.99 to 1.13)	1.23 (1.09 to 1.38)	1.10 (1.04 to 1.17)
10 to 19 yr	1.05 (0.98 to 1.13)	1.10 (0.98 to 1.24)	1.06 (0.99 to 1.14)
≥20 yr	1.00 (0.91 to 1.09)	1.05 (0.92 to 1.19)	1.01 (0.93 to 1.10)

RR, relative risk.

Migraine was dichotomized into migraine versus no migraine.

^a^Adjusted for age.

^b^Adjusted for age and Sleep Problems Index II.

### Shift work duration

Compared with permanent day workers, participants with <10 yr of shift work had a slightly higher prevalence of headache in the combined analyses (RR = 1.04, 95% CI: 1.01 to 1.08). Estimates for participants with 10 to 19 yr and ≥20 yr of shift work were close to unity (Table 2).

For migraine, participants with <10 yr of shift work had a higher prevalence in the combined analyses (RR = 1.10, 95% CI: 1.04 to 1.17), whereas estimates for participants with 10 to 19 yr and ≥20 yr of shift work were closer to unity (Table 3).

### Current shift work

Current shift work was not consistently associated with headache or migraine. In the combined analyses, the prevalence ratio for headache was 0.99 (95% CI: 0.96 to 1.02) in the age-adjusted model and 0.98 (95% CI: 0.96 to 1.01) after additional adjustment for sleep problems (Table 4). Corresponding estimates for migraine were 0.95 (95% CI: 0.90 to 0.99) and 0.94 (95% CI: 0.89 to 0.99), respectively (Table 5).

**Table 4:** Relative risks for headache according to current shift work status estimated using Poisson regression with robust variance.

	Baseline questionnaire (2011)	Follow-up questionnaire (2017)	Combined
	RR (95% CI)	RR (95% CI)	RR (95% CI)
*Current shift work status*			
Model 1^a^			
No current shift work	1.00	1.00	1.00
Current shift work	0.98 (0.95 to 1.01)	1.01 (0.97 to 1.05)	0.99 (0.96 to 1.02)
Model 2^b^			
No current shift work	1.00	1.00	1.00
Current shift work	0.99 (0.96 to 1.02)	0.98 (0.94 to 1.02)	0.98 (0.96 to 1.01)

RR, relative risk.

Headache was dichotomized into headache (light, moderate, or severe) versus no headache.

^a^Adjusted for age.

^b^Adjusted for age and Sleep Problems Index II.

**Table 5:** Relative risks for migraine according to current shift work status estimated using Poisson regression with robust variance.

	Baseline questionnaire (2011)	Follow-up questionnaire (2017)	Combined
	RR (95% CI)	RR (95% CI)	RR (95% CI)
*Current shift work status*			
Model 1^a^			
No current shift work	1.00	1.00	1.00
Current shift work	0.94 (0.89 to 1.00)	0.96 (0.89 to 1.04)	0.95 (0.90 to 0.99)
Model 2^b^			
No current shift work	1.00	1.00	1.00
Current shift work	0.94 (0.88 to 0.99)	0.95 (0.88 to 1.03)	0.94 (0.89 to 0.99)

RR, relative risk.

Migraine was dichotomized into migraine versus no migraine.

^a^Adjusted for age.

^b^Adjusted for age and Sleep Problems Index II.

### Changes in shift work status

Compared with participants who remained permanent day workers, stopping shift work was associated with a higher probability of headache recovery (RR = 1.13, 95% CI: 0.99 to 1.29). No clear associations were found between changes in shift work status and headache recovery or development overall (Table 6).

**Table 6:** Relative risks for headache recovery and development according to changes in current shift work status between baseline and follow-up estimated using Poisson regression with robust variance.

	Headache recovery^a^	Headache development^b^
	RR (95% CI)	RR (95% CI)
*Change in current shift work status*		
Model 1^c^		
Permanent day work	1.00	1.00
Stopped shift work	1.13 (0.99 to 1.29)	1.06 (0.88 to 1.27)
Started shift work	0.97 (0.85 to 1.11)	0.97 (0.82 to 1.14)
Permanent shift work	0.98 (0.90 to 1.06)	0.97 (0.88 to 1.08)

RR, relative risk.

^a^Headache recovery was defined as the transition from headache (light, moderate, or severe) at baseline to no headache at follow-up.

^b^Headache development was defined as the transition from no headache at baseline to headache (light, moderate, or severe) at follow-up.

^c^Adjusted for age.

### Headache and migraine at follow-up stratified by baseline status

Associations between ever shift work and headache or migraine at follow-up were similar among participants with and without headache or migraine at baseline (Table S3).

## Discussion

The findings of this study suggest a small association between ever shift work and migraine, whereas associations with headache were weaker and attenuated after adjustment for sleep problems. Current shift work was not consistently associated with headache or migraine. These findings suggest that the association between shift work and headache disorders may differ between headache and migraine and according to the aspect of shift work exposure considered. Although some associations reached statistical significance, the small magnitude of the observed RRs suggests that their clinical significance at the individual level is likely limited.

The attenuation of the association with headache after adjustment for sleep problems is consistent with the hypothesis that sleep disruption may be relevant in the relationship between shift work and headache disorders. However, sleep problems may be a confounder, an intermediate factor, or a consequence of headache, and our analyses were not designed to distinguish between these possibilities. Therefore, the sleep-adjusted models should be interpreted as exploratory rather than as formal mediation analyses.

Our findings are broadly consistent with previous research suggesting an association between shift work and headache disorders, although reported estimates vary across studies. A recent meta-analysis reported a positive association between shift work and headache across observational studies (Wang et al. 2024). Differences in exposure definitions, outcome assessment, and study design may explain some of the heterogeneity across studies. In our study, headache and migraine were assessed using validated symptom-based instruments, whereas some previous studies relied on open-ended questions, physician diagnoses, treatment-based measures, or healthcare-seeking outcomes.

A systematic review on shift work and migraine likewise reported heterogeneous findings across studies (Leso et al. 2020). Our findings fall within this range, showing a small association between ever shift work and migraine, particularly at follow-up. The review also discussed a cross-sectional study among nurses that reported a higher prevalence of migraine with longer shift exposure (Wang et al. 2015). In our study, the highest prevalence ratios for migraine were observed in the shortest shift work duration category, and estimates were closer to unity among nurses with longer shift work duration. This pattern should be interpreted cautiously, as we did not formally test a dose–response relationship and the duration categories may be affected by selection out of shift work over time.

Our findings are also comparable to those from the Danish PRISME study, which reported higher odds of headache and migraine among shift workers (Appel et al. 2020). However, that study did not evaluate shift work duration, and differences in study populations, exposure definitions, outcome assessment methods, and approaches to confounding control may partly explain why the estimates in our study were small.

Finally, our findings regarding changes in shift work are broadly consistent with results from a Norwegian cohort of nurses, which reported higher odds of headache recovery among workers who reduced shift exposure (Kristoffersen et al. 2024). In our cohort, nurses who stopped working shifts also had a higher probability of headache recovery, supporting the interpretation that changes in work schedules may influence headache outcomes.

### Healthy worker survivor effect

In the present study, estimated prevalence ratios for both headache and migraine were highest among nurses with shorter durations of shift work and were closer to unity among nurses with longer shift work histories. One possible explanation is the healthy worker survivor effect, whereby workers who develop health problems are more likely to leave shift work over time, resulting in a relatively healthier group among those who continue working shifts (Brown et al. 2017). In this context, nurses experiencing recurrent headache or migraine may preferentially transfer to permanent day work, thereby reducing the observed association among long-term shift workers.

Our findings regarding changes in shift work status are compatible with this explanation. Nurses who stopped shift work showed a higher probability of headache recovery than those who remained permanent day workers, although the confidence interval included unity. No clear associations were observed for headache development. Together, these findings provide limited but consistent support for the hypothesis that selection out of shift work may contribute to the observed pattern according to shift work duration.

### Strengths and limitations

The main strengths of this study are its large sample size, with almost 60,000 nurses participating at baseline, and the availability of both baseline and follow-up data. The large study population provided substantial statistical power and represents one of the largest cohorts examining the association between shift work and headache disorders. Furthermore, the use of validated instruments to assess headache and migraine (HIT-6 and ID-Migraine) (Lipton et al. 2003; Yang et al. 2011) strengthens the reliability of the outcome assessment.

Several limitations should be considered. Some analyses were cross-sectional, which limits the ability to determine the temporal relationship between shift work and headache outcomes. Because shift work history and headache were assessed using questionnaires, the timing of symptom onset relative to changes in shift schedules cannot be determined precisely. Shift work exposure was also self-reported, which may have introduced exposure misclassification, particularly for historical exposure patterns. Residual confounding by unmeasured factors, including comorbidities or work-related stress, cannot be excluded.

Furthermore, headache and migraine were assessed using self-administered questionnaires. While these questionnaires have been validated, clinical in-person assessment is generally considered more reliable (Phillip et al. 2007; Yang et al. 2011). Nevertheless, the use of the validated HIT-6 and ID-Migraine instruments is a major strength compared with studies relying on non-validated symptom questions.

Another limitation is the potential for attrition bias due to loss to follow-up. Approximately 37% of participants who completed the baseline questionnaire did not participate in the follow-up. Participants lost to follow-up were somewhat younger and slightly less likely to have a history of shift work, headache, or migraine at baseline. However, standardized differences between responders and non-responders were generally small, suggesting that attrition was unlikely to substantially affect the observed associations, although some bias in the longitudinal analyses cannot be excluded.

Finally, missing data were limited for most variables, although a relatively large proportion of participants had missing information on shift work duration at follow-up. Despite these limitations, our study remains substantially larger than previous studies investigating shift work and headache disorders.

## Conclusion

In this large cohort of Dutch nurses, ever shift work was associated with a small increase in the prevalence of migraine, whereas associations with headache were weaker and attenuated after adjustment for sleep problems. Current shift work was not consistently associated with headache or migraine. Although nurses with shorter durations of shift work generally had higher prevalence ratios than those with longer shift work histories, these findings should be interpreted cautiously and may partly reflect the healthy worker survivor effect. Future longitudinal studies with repeated measurements are needed to further disentangle the relationships between shift work, sleep, and headache disorders.

## Supplementary Material

wxag080_Supplementary_Data

## Data Availability

The data underlying this article will be shared by the authors upon reasonable request.
