# Supplementary material for: Shift work, headache, and migraine in a large cohort of Dutch nurses
Source: Ann Work Expo Health. 2026 Sep 16;70(7):wxag080. doi: 10.1093/annweh/wxag080 (PMC13579698; doi:10.1093/annweh/wxag080)
Supplement: Supplementary file 1 [file wxag080_supplementary_data.pdf]

Supplementary Material

Shift work, headache, and migraine in a large cohort of Dutch nurses

Tara van der Grinten[1*], Sylvia H.J. Jochems[1*], Flora van Leeuwen[2], Michael Schaapveld[2], Hans Kromhout[1], Roel Vermeulen[1], Jelle Vlaanderen[1], Susan Peters[1]

1 Institute for Risk Assessment Sciences, Utrecht University, the Netherlands
2 Department of Epidemiology, Netherlands Cancer Institute, Amsterdam, the Netherlands

*joint first authors

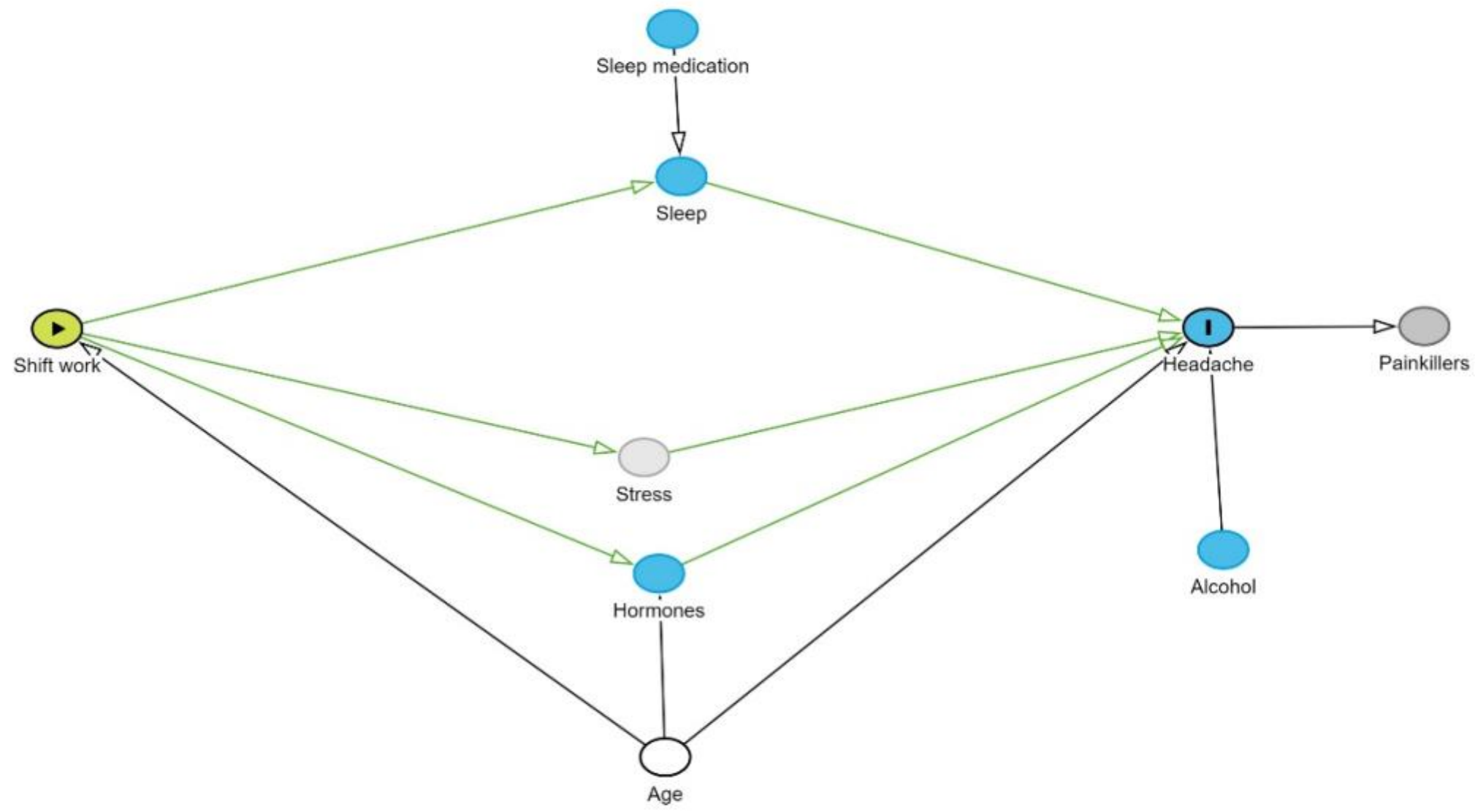

Supplementary Figure 1: Directed Acyclic Graph of the relationship between shift work and headaches

Supplementary Table 1. HIT-6 questionnaire for headache assessment<br>Resulting score of 36 – 78 points, with higher scores indicating more severe headaches					
When you have a headache…					
	Never	Rarely	Sometimes	Very often	All the time
How often is the pain severe?					
How often are you limited in your ability to doing usual daily activities, such as household, work, study/training or social activities?					
How often do you wish you could lie down?					
How often in the past 4 weeks…					
	Never	Rarely	Sometimes	Very often	All the time
Have you felt too tired to go to do work or daily activities because of a headache?					
Did it become too much for you or did you feel irritated because of headache?					
Did a headache limit your ability to concentrate on your work or daily activities?					
Scoring					
Points per answer	6	8	10	11	13

**Supplementary Table 2. ID migraine test**<br><br>Scoring: 1 point per question answered yes. Migraine is defined as scoring 2 or more points		
In the past 3 months, were your headaches accompanied by any of the following?		
	No	Yes
You felt nauseous or had to (almost) vomit		
You were bothered by the light (much more than when you don't have a headache)		
The headache limited your ability to work, study or do what you had to do		

**Supplementary Table 3. Relative risks for headache and migraine at follow-up according to ever shift work, stratified by baseline headache and migraine status, estimated using Poisson regression with robust variance**				
	Headache outcome*		Migraine outcome**	
	No headache at baseline	Headache at baseline	No migraine at baseline	Migraine at baseline
	RR (95% CI)	RR (95% CI)	RR (95% CI)	RR (95% CI)
Ever shift work				
Model 1[a]				
Never shift work	1.00	1.00	1.00	1.00
Ever shift work	1.04 (0.94 – 1.16)	0.99 (0.95 – 1.04)	1.08 (0.92 – 1.26)	1.11 (0.99 – 1.25)
Model 2[b]				
Never shift work	1.00	1.00	1.00	1.00
Ever shift work	1.04 (0.94 – 1.15)	0.98 (0.93 – 1.03)	1.06 (0.91 – 1.24)	1.08 (0.96 – 1.20)

RR = relative risk
*Headache was dichotomized into headache (light, moderate, or severe) versus no headache
**Migraine was dichotomized into migraine versus no migraine
[a] Adjusted for age
[b] Adjusted for age and Sleep Problems Index II
